# Supplementary material for: A virtual intervention to support educator well-being and students’ mental health in conflict-affected Ukraine: A non-randomized controlled trial
Source: Glob Ment Health (Camb). 2025 Jun 5;12:e59. doi: 10.1017/gmh.2025.10014 (PMC12231537; doi:10.1017/gmh.2025.10014)
Supplement: Powell et al. supplementary material [file S2054425125100149sup001.docx]

**Appendix 1: Graphic depiction of change over time**
